# A Wake Up Call for Academic Surgery

**DOI:** 10.1007/s00268-021-06303-0

**Published:** 2021-09-03

**Authors:** Kirsty Mozolowski, Lorna Marson

**Affiliations:** Division of Clinical and Surgical Sciences, University of Edinburgh, Edinburgh, Scotland

There may never be a better opportunity for surgeons to consider how to bring about radical change in order to better represent the public we serve. The paper published by Seehra et al. brings into stark reality the lack of ethnic and gender diversity in presenters at the leading prize sessions of two major surgical conferences in the UK: the Patey prize (Surgical Research Society, SRS) and the Moynihan prize (Association of Surgeons of Great Britain and Ireland, ASGBI). Of 442 presenters over the last 20 years, 211 of them were White males (47.7%), 112 were Asian males (25.3%), and one Black male presented (0.23%); 85 women presented their work (19%), 16 of these women were Asian, and one was Black [1]. These do not represent the numbers of women in surgical training (45%), but lie closer to the percentage of consultant female surgeons currently working in the UK (14%). The percentage of senior female academic surgeons is considerably lower.

This paper does not provide details of the number of women and people of colour who submit abstracts, nor the percentage acceptance rate, but starts with those who have been accepted for the prize sessions. Whether this is a failure of the pipeline or a disparity in the quality of submissions between the different groups is not important: the reality is that women and non-Asian people of colour are not getting the opportunity to showcase their work, which, as the paper demonstrates, has a high chance of being published in peer-reviewed journals, with a median time from presentation to publication of 448 days. The paper does demonstrate that when women do present, they are as likely to win as their male colleagues.

The reasons behind such disparities are complex, as reflected in the report published this year by the Royal College of Surgeons of England [2]. This review of diversity, inclusion, and belonging highlighted some shocking examples of racism and sexism that many of us had hoped were consigned to the past, as well as raising issues of systemic discrimination, differential attainment, and unconscious bias. Much of the criticism focussed on leadership and how the lack of diversity here was both a symptom of the issue and allowed it to persist. Of note, the SRS has had no female Presidents, the ASGBI has had one, Professor Averil Mansfield (1993), and the current President Elect of ASGBI is Professor Gillian Tierney. The paucity of women in prominent leadership roles means that young women who aspire to a career in surgery do not see a clear path to their goal, and may be discouraged, particularly if they are faced with discrimination. Another interesting focus for these societies and others like them is to see how representative are the decision-making panels, the Council and Session Chairs. A lack of diversity in these groups limits perspectives, sends the wrong message to attendees, and fails to offer the opportunity for women and people of colour to obtain recognition and standing [3]. One practical stance that societies such as ASGBI and SRS can make is to commit to avoid male-only committees or panels, or ‘manels’ as they have become known, and to ensure ethnic diversity. In her commentary, Kibbe goes one step further, suggesting that invited panel members refuse to participate in such ‘manels’, thus demonstrating allyship to their poorly represented colleagues.

The authors of the paper by Seehra et al. advocated early exposure to academia in medical school and mentorship of those who have an interest in academic surgery, which is important to inspire and encourage, but if there is a perception of closed doors to those from certain groups further along the career journey, early intervention will not be sufficient to make a difference.

Fitting high-quality research around the rigours of surgical practice is notoriously challenging and changes in working hours have led to the shift of non-clinical work such as research, audit, and exam preparation being pushed into the home lives of surgical trainees [4]. If a surgeon has a demanding home life with caring responsibilities, there is less time available to complete this non-clinical work. Women are most likely to be disadvantaged in this regard given that they assume the majority of domestic and childcare responsibilities, although we should acknowledge the increased workload that all parents and carers have compared to colleagues without. One solution to this is to undertake a dedicated period of research, and this is common in many surgical programmes. This serves to ensure that surgeons have a good understanding of research methodology and evidence-based practice which are commonly part of the criteria to complete surgical training. It is not, however, compulsory to undertake a higher degree and is dependent on the trainee seeking out projects, supervision, and funding. To some this may seem a fairly straightforward process but, given that the academic world suffers from many of the same issues regarding diversity that are present in the surgical world, as evidenced by initiatives such as the Athena Swan and Race Equality Charter, it will be more straightforward for some than others. ‘Like begets like’ is true in the academic and surgical worlds where the make-up of those in leadership positions is often reflected in those who apply for and are recruited to more junior positions [5].

For many years, I have been reassured by senior male colleagues in leadership positions that it will all work out; as more women come through the system, we will be better represented in these senior and leadership positions. This has not borne out: the percentage of female surgical consultants has only increased from 9% in 2012 to 14% in 2020. As stated in the RCS report: ‘lack of diversity is not going to sort itself out over time’, and we must be proactive in addressing these issues if surgery in general, and academic surgery in particular, is to thrive and to inspire the next generation of excellent surgeons, irrespective of race, ethnicity, gender identity, sexual orientation, or socioeconomic background.
